# Open label smoking cessation with varenicline is associated with decreased glutamate levels and functional changes in anterior cingulate cortex: preliminary findings

**DOI:** 10.3389/fphar.2014.00158

**Published:** 2014-07-08

**Authors:** Muriah D. Wheelock, Meredith A. Reid, Harrison To, David M. White, Karen L. Cropsey, Adrienne C. Lahti

**Affiliations:** ^1^Department of Psychiatry and Behavioral Neurobiology, The University of Alabama at BirminghamBirmingham, AL, USA; ^2^Department of Psychology, Behavioral Neuroscience, The University of Alabama at BirminghamBirmingham, AL, USA; ^3^Department of Biomedical Engineering, The University of Alabama at BirminghamBirmingham, AL, USA

**Keywords:** addiction, smoking, varenicline, fMRI, MRS, PPI, connectivity, nicotine

## Abstract

**Rationale:** Varenicline, the most effective single agent for smoking cessation, is a partial agonist at α4β2 nicotinic acetylcholine receptors. Increasing evidence implicates glutamate in the pathophysiology of addiction and one of the benefits of treatment for smoking cessation is the ability to regain cognitive control.

**Objective:** To evaluate the effects of 12-week varenicline administration on glutamate levels in the dorsal anterior cingulate cortex (dACC) and functional changes within the cognitive control network.

**Methods:** We used single-voxel proton magnetic resonance spectroscopy (^1^H-MRS) in the dACC and functional MRI (fMRI) during performance of a Stroop color-naming task before and after smoking cessation with varenicline in 11 healthy smokers (open label design). Using the dACC as a seed region, we evaluated functional connectivity changes using a psychophysiological interaction (PPI) analysis.

**Results:** We observed a significant decrease in dACC glutamate + glutamine (Glx)/Cr levels as well as significant blood oxygen level-dependent signal (BOLD) decreases in the rostral ACC/medial orbitofrontal cortex and precuneus/posterior cingulate cortex. These BOLD changes are suggestive of alterations in default mode network (DMN) function and are further supported by the results of the PPI analysis that revealed changes in connectivity between the dACC and regions of the DMN. Baseline measures of nicotine dependence and craving positively correlated with baseline Glx/Cr levels.

**Conclusions:** These results suggest possible mechanisms of action for varenicline such as reduction in Glx levels in dACC and shifts in BOLD connectivity between large scale brain networks. They also suggest a role for ACC Glx in the modulation of behavior. Due to the preliminary nature of this study (lack of control group and small sample size), future studies are needed to replicate these findings.

## Introduction

Varenicline is an α4β2 nicotinic acetylcholine receptor (nAChR) partial agonist (Rollema et al., 2007) that is more efficacious than nicotine replacement therapies or bupropion for smoking cessation (Gonzales et al., 2006) but still only results in smoking cessation rates of 25–30% 6 months after treatment (Tonstad et al., 2006). Thus, while varenicline is the single best agent currently available, new medications are needed for smoking cessation treatment. Apart from its action at nAChR, little is known about the mechanism of action of this drug. It is critical to understand the mechanism of action of varenicline so that more effective smoking cessation drugs can be developed.

Increasing evidence implicates glutamate in the pathophysiology of addiction (Kalivas et al., 2009), including nicotine addiction (Markou, 2008). Preclinical research in animal models of nicotine-dependence indicates that reducing glutamatergic transmission through pharmacological manipulations decreases the rewarding effect of nicotine (Paterson et al., 2003; Paterson and Markou, 2005). Furthermore, pre-clinical animal research indicates varenicline may act on glutamate levels by increasing glutamic acid decarboxylase expression in prefrontal cortex (Maloku et al., 2011). In humans, measures of central glutamate levels can be obtained using proton magnetic resonance spectroscopy (^1^H-MRS). Specifically, prior MRS studies suggest a role for ACC glutamate levels in the modulation of addiction behaviors. A recent MRS study in alcohol-dependent subjects (Umhau et al., 2010) found a reduction of glutamate levels in the anterior cingulate cortex (ACC) following a 4-week treatment with acamprosate, a drug approved for the maintenance of alcohol dependence. Similarly, a positive correlation was found between alcohol craving and rostral ACC Glx levels in alcohol-dependent patients immediately after detoxification (Bauer et al., 2013). Changes in ACC glutamate after administration of varenicline might suggest a mechanism by which the drug decreases addiction. However, there have been no prior MRS studies of pharmacologically supported smoking cessation.

A number of functional magnetic resonance imaging (fMRI) studies have been conducted investigating the effects of varenicline; they suggest that the drug diminishes physiological responses that lead to relapse such as smoking cue reactivity (Franklin et al., 2011) and the mental disturbances experienced during withdrawal (Loughead et al., 2010; Sutherland et al., 2013). One of the long term benefits of treatment for smoking cessation is the ability to regain control over smoking cues. Several imaging studies indicate that cognitive control, or the ability to shift the processing of perceptual information toward relevant goals, is impaired in addiction (Harding et al., 2012). Azizian et al. (2010) showed that nicotine modulates the blood oxygen level dependent (BOLD) response in regions of the cognitive control network (CCN) (Niendam et al., 2012). On the other hand, investigation of the effects of nicotine on functional connectivity (FC), a measure of temporal coherence of spontaneous neural activity between brain regions, reported changes in FC with regions of the default mode network (DMN) (Raichle and Snyder, 2007), a neural network associated with rest and independent of task or external stimuli (Mason et al., 2007). Since acute administration of nicotine changes the FC between regions of the CCN and DMN (Cole et al., 2010), we were interested in assessing the effect of varenicline on BOLD response and FC between networks.

The aim of this study was to investigate the effect of a 12 week smoking cessation therapy with varenicline on glutamate levels and cognitive control function. We acquired single-voxel ^1^H-MRS measurements in the dorsal ACC (dACC) to obtain glutamate measurements. To activate the CCN, we chose a Stroop color-naming task, which has been shown to produce robust activation in the dACC during high-conflict trials (Azizian et al., 2010). To investigate CCN FC during the Stroop, we conducted a psychophysiological interaction analysis with the dACC as seed region. On the basis of the existing literature (Markou, 2008; Kalivas et al., 2009; Azizian et al., 2010; Cole et al., 2010), we hypothesized that dACC glutamate + glutamine (Glx) levels would decrease and dACC BOLD signal and connectivity would be altered after treatment with varenicline. Finally, to explore patterns that would predict adherence to smoking cessation therapy, we contrasted glutamate levels and BOLD response between those who completed the study and those who dropped out after completing the baseline scanning session.

## Materials and methods

### Participants

We recruited 22 adult participants (age 19–65) who smoked at least 10 cigarettes per day (cpd) and desired smoking cessation treatment via advertisements in flyers and the University of Alabama at Birmingham (UAB) newspaper. Exclusion criteria were major medical conditions, substance abuse other than nicotine within 6 months prior to enrollment, previous serious head injury, a neurologic disorder, loss of consciousness for more than 2 min, and pregnancy. The UAB Institutional Review Board approved the study, and all participants provided written informed consent prior to enrollment.

Participant smoking history, cpd, and measures of nicotine dependence, withdrawal, and craving were assessed weekly for the first 4 weeks and at the 12th week using the Fagerström Test for Nicotine Addiction (FTND) (Heatherton et al., 1991), Minnesota Nicotine Withdrawal Scale (M-NWS; 7 items following DSM-IV criteria for nicotine withdrawal) (Hughes and Hatsukami, 1986), and the Questionnaire of Smoking Urges Short Form (QSU-brief; 10 item measure of craving) (Cox et al., 2001). Common side effects associated with the use of pharmacotherapy and quitting smoking were assessed using the Symptoms and Side Effects questionnaire (SSE; 30 items) (Markou, 2008). Depressive symptoms were assessed using the Center for Epidemiological Studies on Depression Scale (CES-D; 20 item self-report measuring depression) (Radloff, 1977), and the Sensitivity to Punishment and Sensitivity to Reward Questionnaire (SPSRQ) (Torrubia et al., 2001) was administered to determine the valence of external rewards and punishments. Expired air carbon monoxide (CO) concentration in parts per million was measured by a Vitalograph BreathCO monitor (Vitalograph, Inc., Lenxa, KS) once a week for the first 4 weeks as well as prior to the second scan and used in combination with participant cpd self-report to confirm smoking status. Smokers were allowed to smoke up to 1 h before the scanning session to avoid nicotine intoxication or withdrawal.

### Study design and pharmacological intervention

Participants were scanned at baseline and after 12 weeks of treatment with varenicline. Following the first scan, participants completed 4 weekly sessions of a modified version of the individual-based Tobacco Dependence Treatment Program (ACT) (Payne and Crews, 2012) and were given daily manufacturer-recommended dosing of varenicline (Pfizer Global Pharmaceuticals, New York, NY). Dosing schedule consisted of 0.5 mg for the first 3 days, 0.5 mg twice daily for the next 4 days, and 1.0 mg twice daily for the next 12 weeks. Nine participants dropped out after the first scanning session, and two participants dropped out after the first ACT session. Two of the participants who dropped out were excluded from baseline fMRI analyses due to missing behavioral responses during the task, and one revoked consent. Eleven participants remained in the study completing all four ACT sessions. Participant compliance was checked using pill counting on a weekly basis. These 11 participants were scanned within 1 week following the completion of the varenicline regimen.

### Imaging parameters

All imaging was performed on a 3T single channel transmit receive head-only Siemens Magnetom Allegra scanner. fMRI data were acquired using a gradient recalled echo-planar imaging (EPI) sequence (repetition time/echo time [*TR*/*TE*] = 2100/30 ms, flip angle = 70°, field of view = 24 × 24 cm^2^, 64 × 64 matrix, 4-mm slice thickness, 1-mm gap, 26 axial slices). A three-dimensional T1-weighted magnetization prepared rapid acquisition gradient-echo sequence (MP-RAGE) structural scan with 1-mm isotropic voxels was acquired for anatomical reference (*TR*/*TE*/*TI* = 2300/3.93/1100 ms, flip angle = 12°, 256 × 256 matrix).

#### ^1^H-MRS

A ^1^H-MRS voxel was placed in the bilateral dACC as described previously (Reid et al., 2010). Manual shimming was performed to optimize field homogeneity across the voxel, and chemical shift selective pulses were used to suppress the water signal. Spectra were acquired using the point-resolved spectroscopy sequence (PRESS; *TR*/*TE* = 2000/80 ms to optimize the glutamate signal and minimize macromolecule contribution, number of averages = 256, scanning time = 8 min 32 s; voxel size 2.7 × 2 × 1 cm^3^) (Bottomley, 1987). MRS data were analyzed in jMRUI (version 3.0) (see Methods in Reid et al., 2010). Briefly, the model consisted of peaks for *N*-acetyl aspartate (NAA), choline (Cho), creatine (Cr), and three peaks for glutamate + glutamine (Glx). NAA, Glx, and Cho were quantified with respect to Cr. Only Glx peaks are reported and discussed in this study. The MRS voxel was positioned on an individual basis at baseline and week 12 to maximize gray matter based on cortical landmarks. Of the participants who did complete the study, one participant did not have post-varenicline MRS spectra, leaving 10 study completers with MRS spectra pre- and post-varenicline.

#### fMRI

An IFIS-SA system running E-Prime software (1.2; Psychology Software Tools, Pittsburgh, Pennsylvania) was used to control stimulus delivery and to record button press responses and reaction times. Participants performed a version of the Stroop color-naming task as described previously (Reid et al., 2010). Stimuli were three words, “RED,” “GREEN,” or “BLUE,” printed in one of those three colors. Incongruent trials were those in which the word and color were different. Participants were instructed to indicate the color of the word and ignore the meaning of the word. The experiment consisted of three runs of 88 trials. The 3 second trials comprised a word stimulus for 1.5 seconds and a fixation cross for 1.5 seconds. fMRI data analyses were implemented in SPM8 (Wellcome Trust Centre for Neuroimaging). Preprocessing of the fMRI data included slice timing correction, motion correction using ArtRepair (0.5 mm cutoff; CIBSR Stanford University, CA), reslicing to 2-mm isotropic voxels, coregistration to the baseline structural scan, smoothing with a 4 mm full width at half maximum Gaussian kernel, and DARTEL normalization to a group template brain and then standard Montreal Neurological Institute space to facilitate between-subjects comparison (Ashburner, 2007). No subjects were excluded based on movement.

### Statistical analyses

#### Behavior

All behavioral scales and responses were analyzed in PASW Statistics 18 (IBM SPSS Inc., Chicago, IL). Comparisons between groups on smoking and other behavioral measures were done using chi-square and analysis of variance (ANOVA) procedures, as appropriate. Paired *t*-tests were used to examine change across the two time points. The Stroop effect was defined as mean reaction time during congruent trials subtracted from mean reaction time during incongruent trials.

#### MRS

Independent samples *t*-tests were used to assess Glx/Cr at baseline between study completers and study non-completers. A paired samples *t*-test was used to assess the change in Glx/Cr from baseline to week 12 in study completers.

#### fMRI

Statistical tests involved two levels, the single-subject level (fixed effects) and the group level (random effects). At the first level, statistical analyses of fMRI data consisted of a single-subject voxel-by-voxel general linear model. Five conditions were included in the model including incongruent trials, congruent trials, error trials, no response trials, and stimulus repetitions (defined as any trial that was an exact repetition of the previous trial) (Reid et al., 2010). Conditions were convolved with the canonical hemodynamic response function with a temporal derivative. Statistical parametric maps (SPMs) were generated for the contrast of correct incongruent trials minus correct congruent trials (Stroop effect). These contrasts were then used at the second level. One sample *t*-tests were used to assess the group mean differences in the Stroop effect at baseline and after treatment with varenicline. A paired samples *t*-test was used to make comparisons between the two time points. Additionally, CO was added to analyses at the second level as a covariate of no interest to account for differential effects of nicotine. Results were not substantially altered by adding this covariate. All images were set at a statistical significance threshold of *p* < 0.05 with family-wise error (FWE) cluster-level correction. To assess the possibility of type II error due to the small sample size, effect size (Cohen's *d*) was calculated voxel-wise for the paired samples comparisons of fMRI data.

#### Psychophysiological interaction (PPI)

Modulatory interactions using a dACC seed region were assessed using the PPI toolbox in SPM8 (Friston et al., 1997). The PPI analysis consists of a design matrix with three regressors: the “psychological variable” in this case the Stroop effect (correct incongruent trials—correct congruent trials), the “physiological variable” representing the signal time course in the seed region (anterior dACC), and a variable representing the interaction of the two previous terms—the psychophysiological interaction. The anterior dACC was defined as one slice forward from the disappearance of the juncture of the anterior corpus callosum from both hemispheres and the posterior end as the first vertical slice posterior to the anterior commissure. Using this structurally defined region of interest (ROI), a time series from the anterior dACC was extracted from each participant's contrast for the Stroop Effect condition. SPMs were thresholded at *p* < 0.05 to ensure the presence of BOLD signal in all participants. A variable representing the interaction term between each time series and the psychological variable (Stroop effect) was constructed for each participant. Subject-specific contrast images representing functional connectivity of the anterior dACC during Stroop were then entered into a paired samples *t*-test in order to assess differences in connectivity between baseline (pre-varenicline) and week 12 (post varenicline) (see **Figure 2**, **Table 4**). FWE cluster-level correction (*p* < 0.05) was applied to all group-level contrasts.

#### Exploratory analyses

Pearson's correlations were used to assess the relationship between dACC Glx and smoking related measures including nicotine dependence (FTND) and nicotine craving (QSU-sf). Bonferroni correction was applied to all correlations.

#### Study completers vs. study non-completers

We compared study completers and those who dropped out after the baseline scan on baseline characteristics, Glx and BOLD response. *T*-tests were performed to assess differences on smoking parameters and dACC Glx. BOLD differences were assessed during the Stroop effect at baseline using SPM8 (*p* < 0.05 FWE cluster level corrected).

## Results

Seven of the 11 study completers (64%) stopped smoking entirely with the other four participants significantly decreasing their cigarette consumption [pre-varenicline cpd *M* = 27.50, *SD* = 15.00 to post-varenicline cpd *M* = 5.50, *SD* = 6.40, *t*_(3)_ = 3.26, *p* = 0.047]. Study completers had significantly lower craving (QSU-sf) scores at week 12. Symptoms of withdrawal (MNWS), sensitivity to reward (SPSRQ), depression (CESD), and symptoms (SSE) did not significantly change pre- to post-varenicline treatment (Table 1).

**Table 1:** **Study Completers: Behavioral and Clinical Measures**.

**Characteristic**	**Baseline (Pre-drug)**	**Week 12 (Post-drug)**	***t*-value**	***p*-value**
**SMOKING BEHAVIOR**				
CO^*^	19.27 ± 8.48	4.36 ± 5.70	6.71	<0.001
CPD	23.60 ± 9.20	2 ± 4.4	9.01	<0.001
**STROOP TASK BEHAVIOR**				
Congruent RT	799 ± 14	762 ± 9	2.36	0.04
Incongruent RT	956 ± 17	918 ± 13	1.73	0.12
Stroop effect RT	154 ± 8	158 ± 6	−0.29	0.78
Task errors	4.36 ± 2.94	11.27 ± 18.00	−1.24	0.24
CLINICAL MEASURES^**^				
FTND	5.89 ± 2.0	–	–	–
MNWS	9.22 ± 5.61	6.00 ± 6.67	1.04	0.33
QSU-brief	39.11 ± 15.37	11.89 ± 3.52	4.54	0.002
SPSRQ	14.44 ± 5.92	16.44 ± 7.59	−1.73	0.12
CESD	9.67 ± 4.24	9.33 ± 4.64	0.23	0.83
SSE	45.44 ± 12.07	38.67 ± 8.02	1.64	0.14

*CESD, Center for Epidemiological Studies on Depression; CO, carbon monoxide; CPD, cigarettes per day; FTND, Fagerström Test for Nicotine Addiction; MNWS, Minnesota Nicotine Withdrawal Scale (Hughes-Hatsukami Withdrawal Scale); RT, reaction time in milliseconds; SPSRQ, Sensitivity to Punishment and Sensitivity to Reward Questionnaire; SSE, Symptoms and Side Effects; QSU-brief Questionnaire of Smoking Urges Short Form*.

*FTND week 12 scores have been omitted. Two of the seven items on the FTND are related to recent smoking behavior. Because subjects are enrolled in a study to reduce smoking behavior, reduced scores on this measure may reflect behavioral changes and not nicotine dependence per se*.

* *scores imputed for two participants*.

** *scores missing for two participants*.

### Behavioral

There were no significant changes in Stroop effect reaction time nor differences in number of errors pre-to-post varenicline. Reaction time on congruent trials was significantly faster at the post-varenicline scan (Table 1).

### MRS

**Figure 1:**
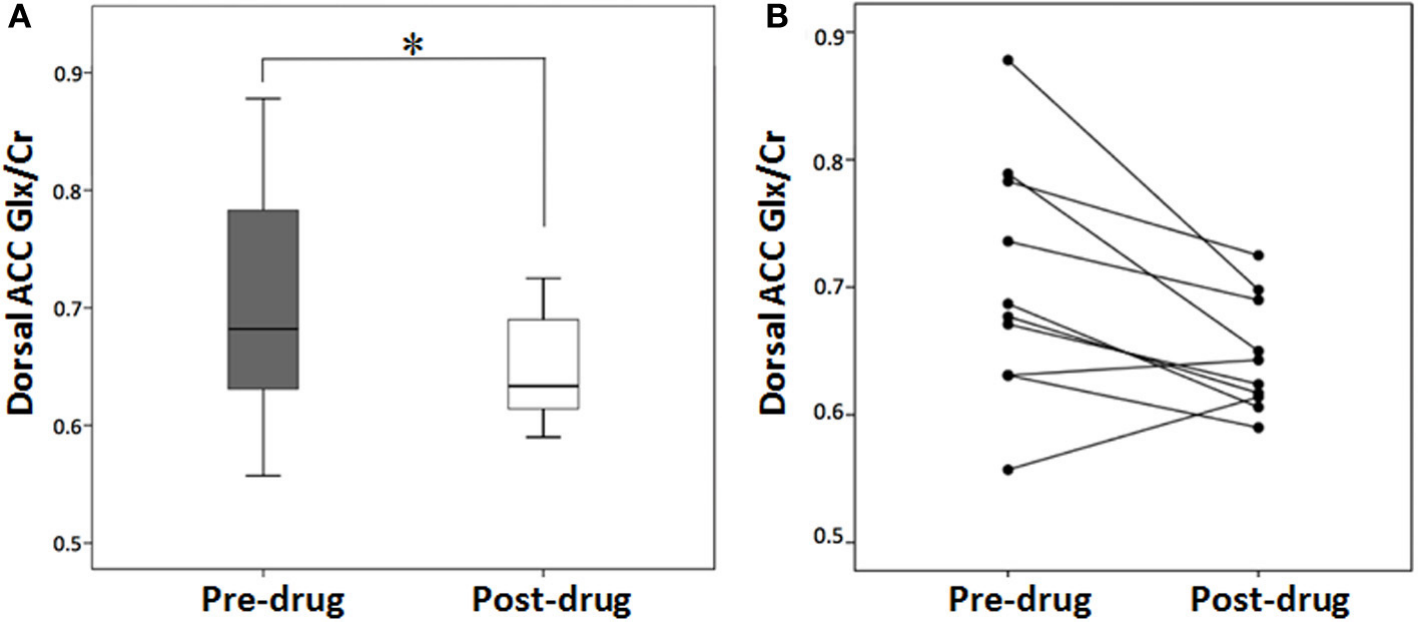
**Dorsal anterior cingulate (dACC), glutamate + glutamine (Glx)/creatine (Cr), baseline (pre-drug), and at week 12 (post-drug) for study completers. (A)** Box plot of median Glx/Cr in the dACC at baseline and week 12. **(B)** Line plot of individual Glx/Cr in the dACC at baseline and week 12. Asterisk indicates significant *t*-statistic at *p* < 0.05.

Study completers had significantly higher Glx/Cr pre-varenicline than post-varenicline (see Table 2, Figures 1A,B). Cr did not significantly differ pre- to post-varenicline *t*_(9)_ = −0.889, *p* = 0.397.

**Table 2:** **Glutamate Measurement in Dorsal Anterior Cingulate Cortex**.

**Metabolite**	**Baseline completers**	**Week 12 completers**	***t*-value**	***p*-value**
Glx/Cr	0.705 ± 0.089	0.646 ± 0.045	2.743	0.023
CRLB ± *SD*	7.1 ± 1.7%	6.9 ± 1.5%		
(CRLB Range)	(5.8–11.6%)	(5.4–10.2%)		

*Cr, creatine; CRLB, Cramer-Rao Lower Bounds; Glx, glutamate + glutamine*.

*There were no significant differences in the raw amplitude of Cr between baseline (pre-drug) and week 12 (post-drug) t_(9)_ = −0.889, p = 0.397. There were no significant differences between baseline to week 12 in metabolite peak line width, CRLBs, gray matter voxel content, or white matter voxel content*.

### fMRI

**Figure 2:**
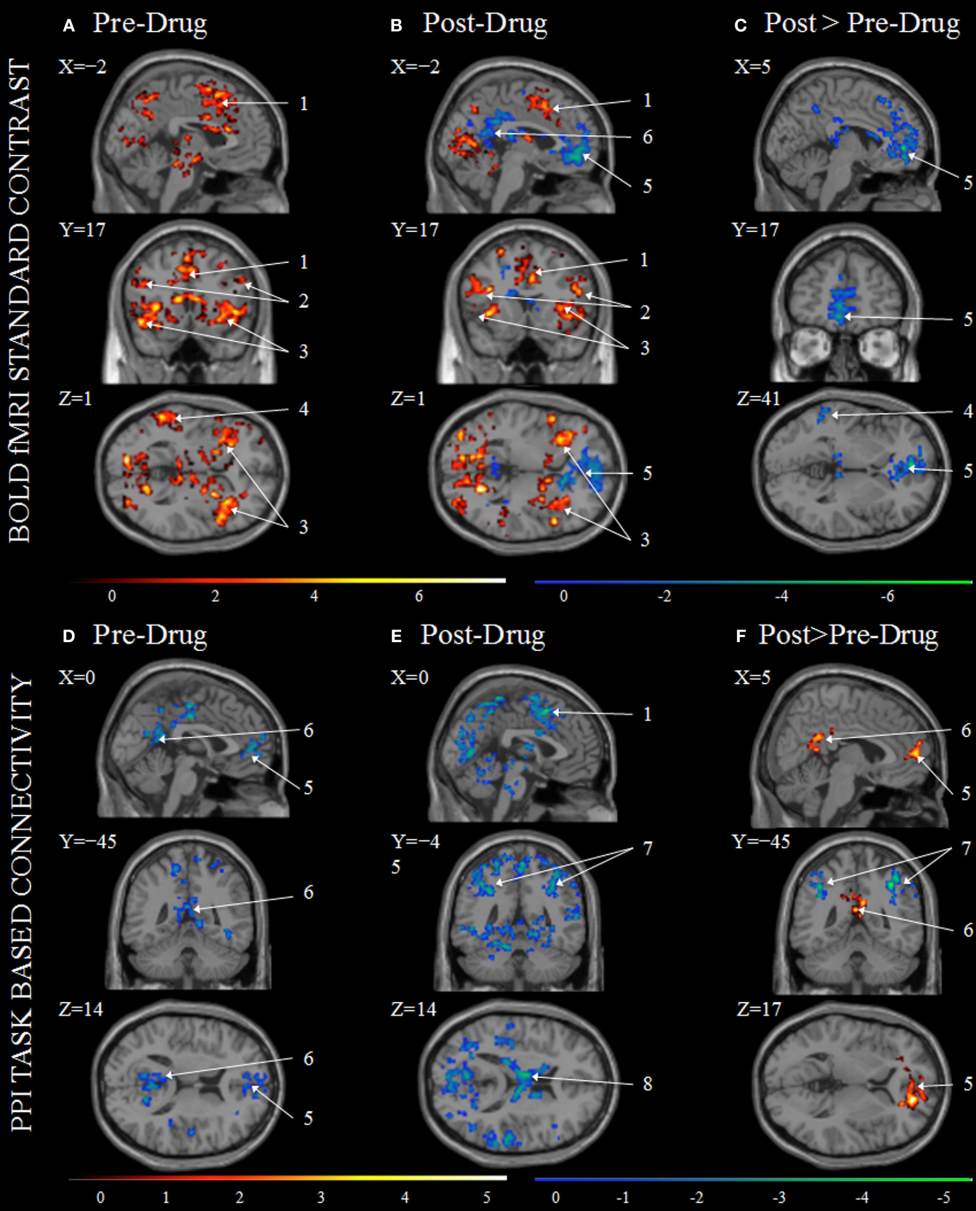
**Stroop task BOLD contrasts and Psychophysiological Interaction (PPI) analysis results**. Group BOLD activation (red) and deactivation (blue) maps shown for BOLD at baseline scans pre-drug **(A)**, BOLD for week 12 scan post-drug **(B)**, and BOLD Post>Pre-drug **(C)** as well as PPI for baseline scan pre-drug **(D)**, PPI post-drug **(E)**, and PPI Post>Pre-drug **(F)**. All color intensities are voxel-wise *t*-statistics with significance *p* < 0.05 FWE cluster-level correction. Activation maps are overlaid onto the averaged anatomical scan Colin27 in MRIcron and are displayed in neurological view (left is on the viewer's left). (1) Supplementary Motor Area (SMA); (2) Inferior Frontal Gyrus; (3) Insula; (4) Middle Temporal Gyrus; (5) rostral ACC connected to medial orbitofrontal cortex; (6) Posterior Cingulate; (7) Inferior Parietal; (8) Thalamus.

Within-group at baseline and week 12 *t*-tests as well as between baseline and week 12 paired *t*-tests of the BOLD during the Stroop Effect are presented in **Figures 2A–C**. Regions including the anterior cingulate, bilateral anterior insula/frontal operculum, right supramarginal/angular gyri, and bilateral dorsolateral prefrontal cortex were identified as significantly activated during incongruent-congruent contrasts in both pre- and post-varenicline scans (**Figures 2A,B**, Table S1). These regions are those typically activated with the Stroop task (Botvinick et al., 1999) and are consistent with activation of the CCN (Niendam et al., 2012). In post-varenicline scans, a significant deactivation can be seen in the rostral ACC extending into the medial orbitofrontal cortex (mOFC), as well as deactivation in the middle cingulate, precuneus, and left temporal lobe (Figure 2B). Deactivation in the post-varenicline scan drove the significant decreased activation in the rostral ACC/mOFC and precuneus seen in the post > pre-varenicline contrast (Figure 2C, Table 3). No regions survived cluster level correction in week 12 > baseline contrasts. Effect size calculation for contrast of post > pre-varenicline showed medium to large effect size across many areas of the brain (Figure S1).

**Table 3:** **Areas with Significant Changes in BOLD Signal for the contrast incongruent greater than congruent following smoking cessation therapy with varenicline**.

**Region**	**Hem**	***x*, *y*, *z***	**Cluster size**	**peak *t*-Value**
**BASELINE>WEEK 12**				
Anterior cingulum	L	−5, 48, 0	840	7.76
**Cluster sub regions**				
Anterior cingulum	R		404	
Middle cingulum	R		229	
Precuneus	R		430	
Caudate	L		203	
Superior frontal	R		159	
Medial sup frontal	L, R		819, 279	
Medial OFC	L, R		324, 152	
Middle temporal	L	−50, −41, 12	339	6.51

*Hem, hemisphere; OFC, Orbitofrontal Cortex; SMA, Supplementary Motor Area; x, y, z refers to Montreal Neurological Institute coordinates. All regions are significant at a cluster level threshold of p < 0.05 FWE cluster level corrected. No regions survived thresholding in Week 12 > Baseline contrast. See Table S1 for within group results*.

### PPI

Voxelwise PPI connectivity with the dACC seed region is presented in Figure 2D. At baseline and week 12, only regions negatively correlated with the dACC seed region survived statistical thresholding (Figures 2D,E). Regions including bilateral superior medial frontal, rostral ACC, precuneus, middle, and posterior cingulate were significantly negatively correlated with the dACC seed region at baseline (Figure 2D). At week 12, dACC was negatively correlated with sensory motor area, bilateral inferior, and superior parietal lobe, precuneus, middle occipital lobe, thalamus, and temporal gyrus (Figure 2E, Table S2). Bilateral medial superior frontal, bilateral rostral ACC, as well as, bilateral posterior and middle cingulum, and bilateral precuneus were significantly more negatively correlated at baseline than at week 12 (Figure 2F, red). Bilateral inferior parietal and right superior occipital were significantly more negatively correlated at week 12 than baseline (Figure 2F blue, Table 4).

**Table 4:** **Changes in dACC functional connectivity for the contrast incongruent greater than congruent following smoking cessation therapy with varenicline**.

**Region**	**Hem**	***x*, *y*, *z***	**Cluster size**	**peak *t*-Value**
**BASELINE>WEEK 12**				
Inferior parietal	R	36, −47, 45	655	7.69
SupraMarginal gyrus	R		279	
Superior occipital	R	33, −72, 42	300	6.37
Angular gyrus	R		252	
Superior parietal	R		237	
Inferior parietal	L	−18, −72, 45	549	5.41
Superior parietal	L		403	
**WEEK 12 > BASELINE**				
Superior medial frontal	R	17, 48, 5	389	7.88
Anterior cingulum	R, L		202, 134	
Superior frontal	R		190	
Superior medial frontal	L		124	
Superior orbitofrontal	R		110	
Posterior cingulum	L	−8, −24, 29	245	5.73
Middle cingulum	L		245	
Precuneus	R, L		175, 170	
Posterior cingulum	R		117	

*Hem, hemisphere; x, y, z refer to Montreal Neurological Institute coordinates. All regions are significant at a cluster level threshold of p < 0.05 FWE cluster level corrected*.

### Exploratory analyses

**Figure 3:**
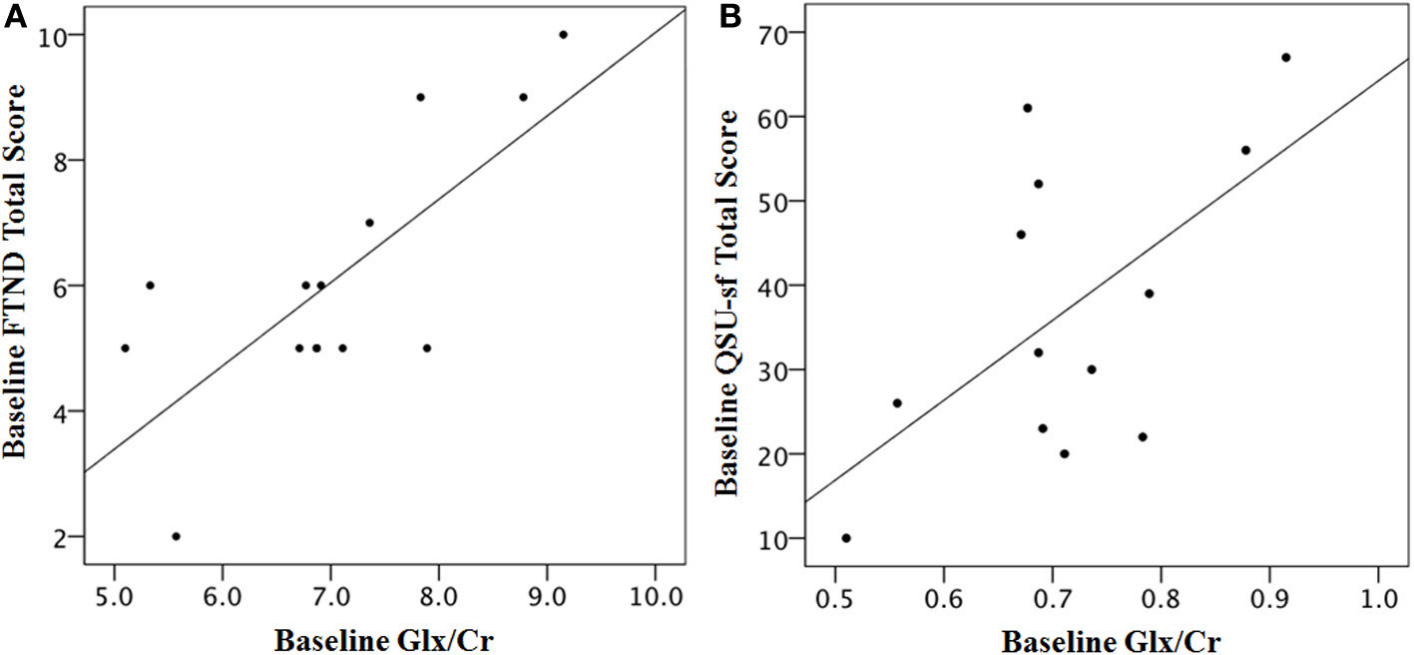
**Baseline scales of nicotine dependence (FTND) and nicotine craving (QSU-sf) correlate with baseline dACC Glx/Cr. (A)** Correlation of Fagerström Test for Nicotine Addiction (FTND) and Glx/Cr: *R*^2^ = 0.558, Pearson *r* = 0.747, *p* = 0.002 two-tailed, significant after Bonferroni Correction **(B)** Questionnaire of Smoking Urges Short Form (QSU-sf), *R*^2^ = 0.458, Pearson *r* = 0.677, *p* = 0.008 two-tailed, significant after Bonferroni Correction. Two completers and five non-completers are missing FTND and QSU-sf data, and one non-completer is missing Glx, leaving a baseline group of 13 participants. FTND and QSU-sf scores were not significantly correlated *r* = 0.378 *p* = 0.165.

A positive correlation was found between baseline nicotine dependence (FTND) and baseline Glx/Cr (Figure 3A) as well as between baseline nicotine craving (QSU-sf) and baseline Glx/Cr (Figure 3B). At baseline, degree of nicotine dependence (Figure 3B) and nicotine craving (Figure 3B) positively correlates with amount of Glx/Cr in the dorsal cingulate. Due to the small sample size and inherent floor effects of the FTND and QSU-sf, post-varenicline nicotine dependence and nicotine craving did not correlate with post-varenicline Glx/Cr.

### Study completers vs. study non-completers

**Figure 4:**
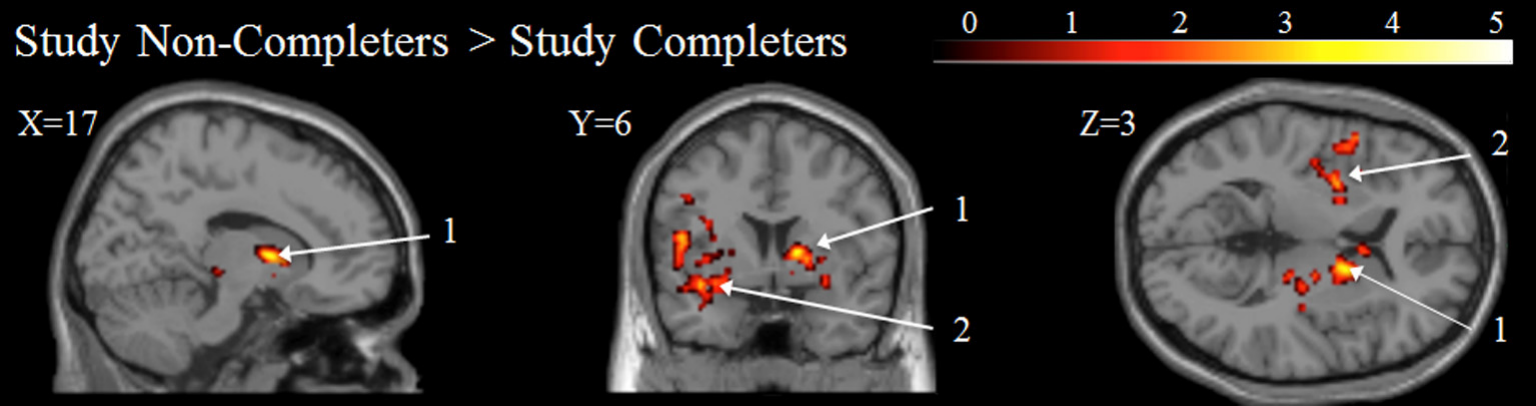
**Baseline BOLD for Study Completers vs. Study Non-completers**. All color intensities are voxel-wise *t*-statistic, *p* < 0.05 FWE cluster-level corrected. Other conventions as in Figure 1. (1) Putamen; (2) Insula.

There were no significant differences between study completers and those who dropped out after the baseline scan on demographics, Stroop effect, smoking behavior, history, or clinical scales (Table 5). Baseline Glx/Cr was not significantly different between study non-completers (*n* = 11) and study completers (*n* = 10) *t*_(19)_ = 0.217, *p* > 0.05 (Table S4). Subcortical regions including the striatum and thalamus, as well as, cortical regions including bilateral insula, left temporal gyrus, and left middle and inferior frontal gyri were significantly more active at baseline in study non-completers than in study completers (Figure 4, Table S3).

**Table 5:** **Baseline Characteristics of Study Participants**.

	**Both groups (***n*** = **22**)**	**Study completers (***n*** = **11**)**	**Study non-completers (***n*** = **11**)**	****t**/χ^2^**	***p*-value**
**DEMOGRAPHICS**					
Gender M, F	10/12	4/7	6/5	0.73	0.39
Race C, AA	17/5	8/3	9/2	0.23	0.61
Age	36.32 ± 11.86	36.0 ± 11.19	36.64 ± 13.03	−0.12	0.90
Education^a^	2.91 ± 0.68	3.09 ± 0.54	2.73 ± 0.79	1.27	0.22
Parental occupation^b^	7.36 ± 5.02	6.0 ± 3.92	8.73 ± 5.78	−1.30	0.21
**SMOKING BEHAVIOR**					
CO^*^	18.87 ± 9.05	19.27 ± 8.48	17.75 ± 11.84	0.28	0.79
CPD	24.60 ± 8.60	23.60 ± 9.20	25.40 ± 8.20	−0.49	0.63
**SMOKING HISTORY^c^**					
Years of smoking	22.46 ± 14.09	18.06 ± 12.67	28.33 ± 14.80	−1.40	0.19
Age daily smoker	17.36 ± 2.74	17.88 ± 2.53	16.67 ± 3.08	0.81	0.44
Prior quit attempts	3.71 ± 3.83	4.88 ± 3.56	2.17 ± 3.92	1.35	0.20
**SMOKING SCALES^d^**					
FTND	5.80 ± 2.27	5.89 ± 2.21	5.67 ± 2.58	0.18	0.86
M-NWS	9.87 ± 6.63	9.22 ± 5.61	10.83 ± 8.42	−0.45	0.66
QSU-brief	33.93 ± 19.19	39.11 ± 15.37	26.17 ± 23.06	1.31	0.21
SPSRQ	14.94 ± 8.28	16.70 ± 9.06	12.0 ± 6.45	1.11	0.29
CESD	9.67 ± 4.69	9.67 ± 4.24	9.67 ± 5.72	0.00	1.00
SSE	40.07 ± 15.60	45.44 ± 12.07	32.0 ± 17.84	1.75	0.10

*AA, African American; C, Caucasian; CESD, Center for Epidemiological Studies on Depression; CO, carbon monoxide; CPD, cigarettes per day; F, Female; FTND, Fagerström Test for Nicotine Addiction; M, Male; M-NWS, Minnesota Nicotine Withdrawal Scale (Hughes-Hatsukami Withdrawal Scale); SPSRQ, Sensitivity to Punishment and Sensitivity to Reward Questionnaire; SSE, Symptoms and Side Effects; QSU-brief, Questionnaire of Smoking Urges Short Form*.

a *Education was scored on a scale from 1 to 4; (1) completed kindergarten through 8th grade; (2) 9th grade through highschool graduate; (3) some college through college graduate; (4) postgraduate education*.

b *Ranks determined from the Diagnostic Interview for Genetic Studies (1–18 scale); higher rank (lower numerical value) corresponds to higher socioeconomic status*.

c *Baseline scores are missing for nine participants*.

d *Responses are missing for seven participants*.

* *Scores imputed for seven study completers and two study non-completers*.

## Discussion

The aim of this study was to evaluate the effects of 12 week varenicline administration on Glx/Cr in the dorsal ACC (dACC) and functional changes within the CCN. Following treatment we observed a significant decrease in dACC Glx/Cr levels, as well as, significant BOLD decreases in the rostral ACC (rACC)/mOFC and precuneus/posterior cingulate cortex (PCC). These BOLD changes are suggestive of alterations in DMN function, and are further supported by the results of the PPI analysis that revealed changes in FC between the dACC and regions of the DMN. Baseline measures of nicotine dependence and craving positively correlated with baseline Glx/Cr levels. Finally, at baseline, study non-completers had significantly more task activation in the ventral striatum, thalamus, and insular cortex than study completers.

To our knowledge there have been no previous MRS studies of pharmacologically supported smoking cessation. As hypothesized, we observed a significant decrease in dACC Glx/Cr levels after treatment (Figure 1A). This 8.7% Glx decrease is in the range of that observed with the use of memantine, an N-methyl-D-aspartate (NMDA) antagonist used for the treatment of Alzheimer's disease (van Wageningen et al., 2010). In a group of active smokers, Gallinat and colleagues found that dACC Glx levels were not significantly different than those of non-smokers or former smokers (Gallinat and Schubert, 2007). These data suggest the Glx decrease seen in this study cannot be interpreted as normalizing Glx levels from an increased state and point toward a drug effect. These results concur with preclinical research showing that reducing glutamatergic transmission through pharmacological manipulations decreases the rewarding effect of nicotine (Markou, 2008) and suggest a mechanism by which varenicline improves abstinence.

To investigate the relationship between glutamate and nicotine addiction we correlated a measure of baseline nicotine dependence (FTND total score) and baseline craving (QSU-sf) with baseline Glx/Cr. Both nicotine dependence and craving were significantly and positively correlated with Glx levels (Figures 3A,B). Interestingly, positive correlations between craving and rostral ACC Glx levels in alcohol-dependent patients immediately after detoxification (Bauer et al., 2013) and between impulsivity and dorsal ACC Glx (Hoerst et al., 2010) have been reported. Collectively, these data suggest a role for ACC glutamate in the modulation of behavior. In addition, thalamic Glx/Cr was found to be higher in patients suffering from restless legs syndrome (RLS) (Allen et al., 2013), and case report found RLS amelioration with varenicline treatment (Romigi and Vitrani, 2013), suggesting a potential mechanism whereby varenicline may reduce thalamic Glx levels in these patients.

After treatment, we observed significant BOLD decreases in rACC/mOFC and precuneus/PCC (Figure 2C). These results show similarities to previous imaging studies with varenicline. After 3 days of nicotine abstinence, while on varenicline, working memory-related brain activity was increased, especially at higher task difficulty, and was associated with improved cognitive performance (Loughead et al., 2010). Interestingly, a whole-brain analysis of this study revealed enhanced deactivations of PCC and rACC in association with varenicline. In non-abstinent smokers, 3 weeks of varenicline administration resulted in a decreased response to smoking cues in the ventral striatum and mOFC (Franklin et al., 2011). Further, abstinent smokers and non-smokers demonstrated decreased resting state DMN connectivity with the insula after 17 days of varenicline (Sutherland et al., 2013). Thus, similar to our results, these studies found alterations in regions of the DMN in association with smoking cessation with varenicline. In this study, these alterations were observed in the face of unchanged task performance.

We evaluated changes in FC during task performance using PPI analysis. Prior to treatment, there was a significant negative FC between the dACC seed and the rACC and PCC, both regions of the DMN (Figure 2D). Activities in the CCN and the DMN have been shown to be negatively related (Fox et al., 2005) so that during task performance the DMN is deactivated in proportion to CCN activation. The loss of this negative coupling appears to interfere with proper task performance (Sonuga-Barke and Castellanos, 2007) while greater negative coupling is usually regarded as advantageous in allocating cognitive resources during task (Kelly et al., 2008). Our pre-varenicline results replicate previous findings of negative functional coupling between the CCN and DMN regions.

Recent studies have reported greater deactivation of the DMN with acute nicotine administration that was associated with improved task performance (Hahn et al., 2007; Ettinger et al., 2009). Furthermore, during resting state fMRI, acute nicotine administered to abstinent smokers increased the negative FC between CCN and DMN networks, an effect associated with improvement in withdrawal symptoms (Cole et al., 2010). In this study, after varenicline, the negative FC between the CCN seed and the DMN was reduced while there was an increased negative FC between the seed and the bilateral inferior parietal cortex (Figure 2F). Increased connectivity between a dACC seed region and parieto-occipital cortex has been found to distinguish cannabis users from controls in an emotional color word Stroop (Harding et al., 2012). The parieto-occipital attention network plays a role in selecting relevant objects and directing appropriate behaviors (Hopfinger et al., 2000). The role of the dACC has been ascribed to direct attention based on behaviorally relevant stimuli (Weissman et al., 2005). Thus, increased negative FC between dACC and regions of the fronto-parietal network may suggest changes in attentional bias toward drug related stimuli. In contrast to studies of the acute effects of nicotine on the DMN (Hahn et al., 2007; Ettinger et al., 2009), the changes in FC reported here were observed after treatment, at a time when participants did not experience symptoms of withdrawal or craving, and most of them had stopped smoking. Based on these observations, we have hypothesized that changes in brain network dynamics are key to understanding the mechanism of sustained action of antismoking drugs.

Nicotine addiction has been linked to its ability to release dopamine through activation of α4β2 nAChRs located on pre-synaptic neurons of the mesolimbic dopamine system (Benowitz, 2008). Smoking-induced increase in dopamine release produces changes in synaptic plasticity (Kalivas and O'Brien, 2008) that reinforce smoking behavior (Fiore et al., 2008) a process leading to addiction. Thus, it is possible varenicline affects the rACC and mOFC through its action on mesolimbic dopaminergic neurons. Speculatively, BOLD changes in these regions could trigger concomitant changes in other regions of the DMN, including the PCC.

While Mashhoon et al. (2011) found that higher Glx/Cr in smokers predicted those who would relapse into smoking, we did not find that Glx/Cr differentiated study completers and non-completers. Perhaps one of the more interesting findings from this study is that treatment completers and non-completers did not differ in demographic and clinical characteristics, but did differ in that non-completers had increased baseline fMRI BOLD intensities in the putamen and insula (Figure 4). Such a finding could be an important clue for future work to help clarify the neurobiological substrates of treatment failure in smoking cessation, especially since these findings appear to be better predictors than any clinical or demographic information available. Converging evidence links the ventral striatum to reward processing (Koob and Volkow, 2010), our results point toward differences in activation of the reward system between the two groups. The role of the insula has been linked to the monitoring of internal bodily states and to the processing of subjective feelings (Craig, 2002, 2009). The insula and connected networks appear to play a critical role in addiction, including nicotine addiction (Naqvi et al., 2007; Sutherland et al., 2012). Importantly, damage to the insula, such as in stroke, can disrupt nicotine addiction (Naqvi et al., 2007). In addition, activity in the insula correlates with cue-induced drug urges (Brody et al., 2002).

Our study has several limitations. The study was not placebo controlled, and the design did not allow disentangling the effects of smoking cessation and varenicline. This study design also did not allow an assessment of potential practice effects due to participants completing the Stroop again, 12 weeks later. Because cerebral blood flow (CBF) can influence the BOLD signal (Kim and Ogawa, 2012), and because varenicline alters CBF (Franklin et al., 2011), it is possible that the observed changes in BOLD activation and connectivity were influenced by changes in CBF. While this study controlled for false positive rates in the fMRI results with FWE correction, the small sample size may lead to false negatives. Furthermore, the results should be considered as preliminary given the small sample size, the number of dropouts, and single-arm open label design of the study. Therefore, these fMRI findings should be confirmed in additional studies in a larger sample with a double blind randomized control trial with smokers and non-smokers. Further studies are needed to understand the relationship of varenicline to glutamate and brain networks dynamics. The quantification of glutamate with ^1^H-MRS is difficult. Glutamate, glutamine (the major metabolite of synaptic glutamate), and GABA have overlapping peaks that are difficult to resolve at low magnetic field strength. A single peak termed Glx containing the three peaks is usually reported. The Glx peak cannot be simply equated with glutamatrgic neurotransmission as extracellular and vesicular glutamate represents only a small fraction of total brain glutamate. A technical strength of our study that partially compensates for this is the use of an echo time of 80 ms which appears to optimize the glutamate signal by diminishing macromolecule contribution and reducing glutamine contribution to the spectra (Bottomley, 1987; Schubert et al., 2004). Finally, the comparisons between completers vs. non-completers were based on small groups.

In conclusion, the results of this study suggest two possible mechanisms of action for varenicline: reduction in glutamate levels in ACC and shifts in BOLD activities between large scale brain networks. Understanding the mechanism of action of antismoking drugs is key to identifying novel and more efficacious compounds for smoking cessation. These findings are preliminary. Further studies are needed to assess the definitive mechanisms of varenicline.

### Conflict of interest statement

The corresponding author received medication and investigator-initiated financial support provided by Pfizer. Pfizer provided no input into the design, collection, analysis, or interpretation of the data, or writing of the manuscript. Pfizer did provide approval of a poster presented at a local conference (UAB-AU Neuroimaging retreat 2012). The information approved by Pfizer for the poster is also found in this manuscript. The authors declare that the research was conducted in the absence of any commercial or financial relationships that could be construed as a potential conflict of interest.

## References

[B1] Allen R. P. Barker P. B. Horska A. Earley C. J. (2013). Thalamic glutamate/glutamine in restless legs syndrome: increased and related to disturbed sleep. Neurology 80, 2028–2034 10.1212/WNL.0b013e318294b3f6 23624560 PMC3716406

[B3] Ashburner J. (2007). A fast diffeomorphic image registration algorithm. Neuroimage 38, 95–113 10.1016/j.neuroimage.2007.07.007 17761438

[B4] Azizian A. Nestor L. J. Payer D. Monterosso J. R. Brody A. L. London E. D. (2010). Smoking reduces conflict-related anterior cingulate activity in abstinent cigarette smokers performing a Stroop task. Neuropsychopharmacology 35, 775–782 10.1038/npp.2009.186 19907418 PMC2813980

[B5] Bauer J. Pedersen A. Scherbaum N. Bening J. Patschke J. Kugel H. (2013). Craving in alcohol-dependent patients after detoxification is related to glutamatergic dysfunction in the nucleus accumbens and the anterior cingulate cortex. Neuropsychopharmacology 38, 1401–1408 10.1038/npp.2013.45 23403696 PMC3682141

[B6] Benowitz N. L. (2008). Clinical pharmacology of nicotine: implications for understanding, preventing, and treating tobacco addiction. Clin. Pharmacol. Ther. 83, 531–541 10.1038/clpt.2008.3 18305452

[B7] Bottomley P. A. (1987). Spatial localization in NMR spectroscopy *in vivo*. Ann. N.Y. Acad. Sci. 508, 333–348 3326459 10.1111/j.1749-6632.1987.tb32915.x

[B8] Botvinick M. Nystrom L. E. Fissell K. Carter C. S. Cohen J. D. (1999). Conflict monitoring versus selection-for-action in anterior cingulate cortex. Nature 402, 179–181 10647008 10.1038/46035

[B9] Brody A. L. Mandelkern M. A. London E. D. Childress A. R. Lee G. S. Bota R. G. (2002). Brain metabolic changes during cigarette craving. Arch. Gen. Psychiatry 59, 1162–1172 10.1001/archpsyc.59.12.1162 12470133

[B10] Cole D. M. Beckmann C. F. Long C. J. Matthews P. M. Durcan M. J. Beaver J. D. (2010). Nicotine replacement in abstinent smokers improves cognitive withdrawal symptoms with modulation of resting brain network dynamics. Neuroimage 52, 590–599 10.1016/j.neuroimage.2010.04.251 20441798

[B11] Cox L. S. Tiffany S. T. Chirsten A. G. (2001). Evaluation of the brief questionnaire of smoking urges (QSU-brief) in laboratory and clinical. Nicotine Tob. Res. 3, 7–16 10.1080/14622200020032051 11260806

[B12] Craig A. D. (2002). How do you feel? Interoxeption: the sense of the physiological condition of the body. Nat. Rev. Neurosci. 3, 655–666 10.1038/nrn894 12154366

[B13] Craig A. D. (2009). Emotional moments across time: a possible neural basis for time perception in the anterior insula. Philos. Trans. R. Soc. Lond. B Biol. Sci. 364, 1933–1942 10.1098/rstb.2009.0008 19487195 PMC2685814

[B14] Ettinger U. Williams S. C. Patel D. Michel T. M. Nwaigwe A. Caceres A. (2009). Effects of acute nicotine on brain function in healthy smokers and non-smokers: estimation of inter-individual response heterogeneity. Neuroimage 45, 549–561 10.1016/j.neuroimage.2008.12.029 19159693

[B15] Fiore M. C. Jaen C. R. Baker T. B. Baily W. C. Benowitz N. L. Curry S. J. (2008). Treating Tobacco Use and Dependence: 2008 Update. Rockvill, MD: US Department of Healthy and Human Services; Public Health Service

[B16] Fox M. D. Snyder A. Z. Vincent J. L. Corbetta M. Van Essen D. C. Raichle M. E. (2005). The human brain is intrinsically organized into dynamic, anticorrelated functional networks. Proc. Natl. Acad. Sci. U.S.A. 102, 9673–9678 10.1073/pnas.0504136102 15976020 PMC1157105

[B17] Franklin T. Wang Z. Suh J. J. Hazan R. Cruz J. Li Y. (2011). Effects of varenicline on smoking cue-triggered neural and craving responses. Arch. Gen. Psychiatry 68, 516–526 10.1001/archgenpsychiatry.2010.190 21199958 PMC3743240

[B18] Friston K. J. Buechel C. Fink G. R. Morris J. Rolls E. Dolan R. J. (1997). Psychophysiological and modulatory interactions in neuroimaging. Neuroimage 6, 218–229 9344826 10.1006/nimg.1997.0291

[B19] Gallinat J. Schubert F. (2007). Regional cerebral glutamate concentrations and chronic tobacco consumption. Pharmacopsychiatry 40, 64–67 10.1055/s-2007-970144 17447175

[B20] Gonzales D. Rennard S. I. Nides M. Oncken C. Azoulay S. Billing C. B. (2006). Varenicline, an α4β 2 nicotinic acetylcholine receptor partial agonist, vs sustained- release bupropion and placebo for smoking cessation. JAMA 296, 47–55 10.1001/jama.296.1.47 16820546

[B21] Hahn B. Ross T. J. Yang Y. Kim I. Huestis M. A. Stein E. A. (2007). Nicotine enhances visuospatial attention by deactivating areas of the resting brain default network. J. Neurosci. 27, 3477–3489 10.1523/JNEUROSCI.5129-06.2007 17392464 PMC2707841

[B22] Harding I. H. Solowij N. Harrison B. J. Takagi M. Lorenzetti V. Lubman D. I. (2012). Functional connectivity in brain networks underlying cognitive control in chronic cannabis users. Neuropsychopharmacology 37, 1923–1933 10.1038/npp.2012.39 22534625 PMC3376324

[B23] Heatherton T. F. Kozlowski L. T. Frecker R. C. Fagerström K. (1991). The Fagerström Test for Nicotine Dependence: a revision of the Fagerström Tolerance Questionnaire. Br. J. Addict. 86, 1119–1127 1932883 10.1111/j.1360-0443.1991.tb01879.x

[B24a] Hoerst M. Weber-Fahr W. Tunc-Skarka N. Ruf M. Schmahl C. Ende G. (2010). Correlation of glutamate levels in the anterior cingulate cortex with self-reported impulsivity in patients with borderline personality disorder and healthy controls. Arch. Gen. Psychiatry 67, 946–954 10.1001/archgenpsychiatry.2010.93 20603446

[B24] Hopfinger J. B. Buonocore M. H. Mangun G. R. (2000). The neural mechanisms of top-down attentional control. Nat. Neurosci. 3, 284–291 10.1038/72999 10700262

[B25] Hughes J. R. Hatsukami D. (1986). Signs and symptoms of tobacco withdrawal. Arch. Gen. Psychiatry 43, 289–294 3954551 10.1001/archpsyc.1986.01800030107013

[B26] Kalivas P. W. Lalumiere R. T. Knackstedt L. Shen H. (2009). Glutamate transmission in addiction. Neuropharmacology 56(Suppl. 1), 169–173 10.1016/j.neuropharm.2008.07.011 18675832 PMC3280337

[B27] Kalivas P. W. O'Brien C. (2008). Drug addiction as a pathology of staged neuroplasticity. Neuropsychopharmacology 33, 166–180 10.1038/sj.npp.1301564 17805308

[B28] Kelly A. M. Uddin L. Q. Biswal B. B. Castellanos F. X. Milham M. P. (2008). Competition between functional brain networks mediates behavioral variability. Neuroimage 39, 527–537 10.1016/j.neuroimage.2007.08.008 17919929

[B29] Kim S. G. Ogawa S. (2012). Biophysical and physiological origins of blood oxygenation level-dependent fMRI signals. J. Cereb. Blood Flow Metab. 32, 1188–1206 10.1038/jcbfm.2012.23 22395207 PMC3390806

[B30] Koob G. F. Volkow N. D. (2010). Neurocircuitry of addiction. Neuropsychopharmacology 35, 217–238 10.1038/npp.2009.110 19710631 PMC2805560

[B31] Loughead J. Ray R. Wileyto E. P. Ruparel K. Sanborn P. Siegel S. (2010). Effects of the alpha4beta2 partial agonist varenicline on brain activity and working memory in abstinent smokers. Biol. Psychiatry 67, 715–721 10.1016/j.biopsych.2010.01.016 20207347

[B32] Maloku E. Kadriu B. Zhubi A. Dong E. Pibiri F. Satta R. (2011). Selective alpha4beta2 nicotinic acetylcholine receptor agonists target epigenetic mechanisms in cortical GABAergic neurons. Neuropsychopharmacology 36, 1366–1374 10.1038/npp.2011.21 21368748 PMC3096806

[B33] Markou A. (2008). Review. Neurobiology of nicotine dependence. Philos. Trans. R. Soc. Lond. B Biol. Sci. 363, 3159–3168 10.1098/rstb.2008.0095 18640919 PMC2607327

[B34] Mashhoon Y. Janes A. C. Jensen J. E. Prescot A. P. Pachas G. Renshaw P. F. (2011). Anterior cingulate proton spectroscopy glutamate levels differ as a function of smoking cessation outcome. Prog. Neuropsychopharmacol. Biol. Psychiatry. 35, 1709–1713 10.1016/j.pnpbp.2011.05.006 21616118 PMC3303218

[B35] Mason M. F. Norton M. I. Van Horn J. D. Wegner D. M. Grafton S. T. Macrae C. N. (2007). Wandering minds: the default network and stimulus - independent thought. Science 315, 393–395 10.1126/science.1131295 17234951 PMC1821121

[B37] Naqvi N. H. Rudrauf D. Damasio H. Bechara A. (2007). Damage to the insula disrupts addiction to cigarette smoking. Science 315, 531–534 10.1126/science.1135926 17255515 PMC3698854

[B38] Niendam T. A. Laird A. R. Ray K. L. Dean Y. M. Glahn D. C. Carter C. S. (2012). Meta-analytic evidence for a superordiante cognitive control network subserving diverse executive functions. Cogn. Affect. Behav. Neurosci. 12, 241–268 10.3758/s13415-011-0083-5 22282036 PMC3660731

[B39] Paterson N. E. Markou A. (2005). The metabotropic glutamate receptor 5 antagonist MPEP decreased break points for nicotine, cocaine and food in rats. Psychopharmacology (Berl.) 179, 255–261 10.1007/s00213-004-2070-9 15619120

[B40] Paterson N. E. Semenova S. Gasparini F. Markou A. (2003). The mGluR5 antagonist MPEP decreased nicotine self-administration in rats and mice. Psychopharmacology (Berl.) 167, 257–264 10.1007/s00213-003-1432-z 12682710

[B41] Payne T. J. Crews K. M. (2012). Brief Treatment of the Tobacco Dependent Patient: A Training Program for Healthcare Providers. Jackson, MS: University of Mississippi Medical Center Printing Office

[B42] Radloff L. S. (1977). The CES-D Scale: a self-report depression scale for research in the general population. Appl. Pyschol. Meas. 1, 385–401

[B43] Raichle M. E. Snyder A. Z. (2007). A default mode of brain function: a brief history of an evolving idea. Neuroimage 37, 1083–1090 discussion: 1097–1089. 10.1016/j.neuroimage.2007.02.041 17719799

[B44] Reid M. A. Stoeckel L. E. White D. M. Avsar K. B. Bolding M. S. Akella N. S. (2010). Assessments of function and biochemistry of the anterior cingulate cortex in schizophrenia. Biol. Psychiatry 68, 625–633 10.1016/j.biopsych.2010.04.013 20570244 PMC2953853

[B45] Rollema H. Chambers L. K. Coe J. W. Glowa J. Hurst R. S. Lebel L. A. (2007). Pharmacological profile of the alpha4beta2 nicotinic acetylcholine receptor partial agonist varenicline, an effective smoking cessation aid. Neuropharmacology 52, 985–994 10.1016/j.neuropharm.2006.10.016 17157884

[B46] Romigi A. Vitrani G. (2013). Improvement of restless legs syndrome by varenicline as antismoking treatment. J. Clin. Sleep Med. 9, 1089–1090 10.5664/jcsm.3092 24127155 PMC3778182

[B47] Schubert F. Gallinat J. Seifert F. Rinneberg H. (2004). Glutamate concentrations in human brain using single voxel proton magnetic resonance spectroscopy at 3 Tesla. Neuroimage 21, 1762–1771 10.1016/j.neuroimage.2003.11.014 15050596

[B48] Sonuga-Barke E. J. Castellanos F. X. (2007). Spontaneous attentional fluctuations in impaired states and pathological conditions: a neurobiological hypothesis. Neurosci. Biobehav. Rev. 31, 977–986 10.1016/j.neubiorev.2007.02.005 17445893

[B49] Sutherland M. T. Carroll A. J. Salmeron B. J. Ross T. J. Hong L. E. Stein E. A. (2013). Down-regulation of amygdala and insula functional circuits by varenicline and nicotine in abstinent cigarette smokers. Biol. Psychiatry 74, 538–546 10.1016/j.biopsych.2013.01.035 23506999 PMC3775982

[B50] Sutherland M. T. McHugh M. J. Pariyadath V. Stein E. A. (2012). Resting state functional connectivity in addiction: lessons learned and a road ahead. Neuroimage 62, 2281–2295 10.1016/j.neuroimage.2012.01.117 22326834 PMC3401637

[B51] Tonstad S. Tonnesen P. Hajek P. Williams K. E. Billing C. B. Reeves K. R. (2006). Effect of maintenance therapy with varenicline on smoking cessation: a randomized controlled trial. JAMA 296, 64–71 10.1001/jama.296.1.64 16820548

[B52] Torrubia R. Avila C. Molto J. Caseras X. (2001). The Sensitivity to Punishment and Sensitivity to Reward Questionnaire (SPSRQ) as a meausre of Bray's anxiety and impulsivity dimensions. Pers. Individ. Dif. 31, 837–862 10.1016/S0191-8869(00)00183-5

[B53] Umhau J. C. Memenan R. Schwandt M. L. Singley E. Lifshitz M. Doty L. (2010). Effect of acamprosate on magnetic resonance spectroscopy measures of central glutamate in detoxified alcohol-dependent individuals. Arch. Gen. Psychiatry 67, 1070–1077 10.1001/archgenpsychiatry.2010.125 20921123 PMC3583213

[B54] van Wageningen H. Jorgensen H. A. Specht K. Hugdahl K. (2010). A 1H-MR spectroscopy study of changes in glutamate and glutamine (Glx) concentrations in frontal spectra after administration of memantine. Cereb. Cortex 20, 798–803 10.1093/cercor/bhp145 19605521

[B55] Weissman D. H. Gopalakrishnan A. Hazlett C. J. Woldorff M. G. (2005). Dorsal anterior cingulate cortex resolves conflict from distracting stimuli by boosting attention toward relevant events. Cereb. Cortex 15, 229–237 10.1093/cercor/bhh125 15238434

