# Supplementary material for: Open label smoking cessation with varenicline is associated with decreased glutamate levels and functional changes in anterior cingulate cortex: preliminary findings
Source: Front Pharmacol. 2014 Jul 8;5:158. doi: 10.3389/fphar.2014.00158 (PMC4085720; doi:10.3389/fphar.2014.00158)
Supplement: Supplementary file 1 [file DataSheet1.DOC]

**Table S1**. Regional labels of BOLD activation and deactivation during Stroop task.

BA, Brodmann’s Area; Hem, hemisphere; L,R indicates bilateral activation with corresponding cluster sizes separated by a comma; OFC, Orbitofrontal Cortex; SMA, Supplementary Motor Area; x,y,z refer to Montreal Neurological Institute coordinates. All regions are significant at a cluster level threshold of p<.05 FWE cluster level corrected.

| **Region** | **Hem** | **BA** | **x,y,z** | **Cluster size** | ***peak t* Value** |
| --- | --- | --- | --- | --- | --- |
| **Baseline Activation** |  |  |  |  |  |
| Primary Motor | R | 9 | 53, 20,30 | 432 | 5.70 |
| Inferior Opercular Frontal | R |  |  | 372 |  |
| Primary Motor | L | 6 | -36,3,45 | 1462 | 14.07 |
| *Cluster Sub Regions* |  |  |  |  |  |
| SMA | L,R |  |  | 999, 927 |  |
| Superior Frontal | L |  |  | 455 |  |
| Middle Frontal | L |  |  | 1023 |  |
| Inferior Triangular Frontal | L |  |  | 1332 |  |
| Inferior Opercular Frontal | L,R |  |  | 609, 345 |  |
| OFC | L |  |  | 298 |  |
| Insula | L,R |  |  | 1153, 1074 |  |
| Middle Cingulum | R |  |  | 555 |  |
| Anterior Cingulum | R |  |  | 370 |  |
| Putamen | L,R |  |  | 472, 327 |  |
| Caudate | R |  |  | 266 |  |
| Inferior Parietal | L | 7 | -38,-56,48 | 2353 | 12.05 |
| *Cluster Sub Regions* |  |  |  |  |  |
| Inferior Parietal | R |  |  | 1120 |  |
| Superior Parietal | L,R |  |  | 1173, 358 |  |
| Angular Gyrus | R |  |  | 586 |  |
| Precuneus | R,L |  |  | 898, 523 |  |
| SupraMarginal Gyrus | R |  |  | 468 |  |
| Superior Occipital | L,R |  |  | 515, 403 |  |
| Middle Occipital | R, L |  |  | 854, 537 |  |
| Calcarine | L | 18 | -12,-86,2 | 606 | 7.78 |
| Calcarine | R |  |  | 379 |  |
| Lingual | R,L |  |  | 284, 281 |  |
| Fusiform | L | 37 | -26,-26,-23 | 653 | 7.01 |
| Inferior Temporal | L |  |  | 256 |  |
| Hippocampus | R | 28 | 45,-23,-17 | 239 | 6.82 |
| Middle Temporal | L | 22 | -51,-41,14 | 744 | 6.24 |
| **Baseline Deactivation** |  |  |  |  |  |
| SMA | R | 6 | 12,-26,54 | 122 | 6.81 |
| **Week 12 Activation** |  |  |  |  |  |
| Middle Occipital  *Cluster Sub Regions* | L | 19 | 18,-63,3 | 1081 | 17.64 |
| Calcarine | L,R |  |  | 919, 808 |  |
| Lingual | L,R |  |  | 665, 423 |  |
| Fusiform | L |  |  | 499 |  |
| Inferior Occipital | L |  |  | 467 |  |
| Cerebellum | L |  |  | 417 |  |
| Middle Temporal | L |  |  | 344 |  |
| Primary Motor  *Cluster Sub Regions* | R |  | 54,6,14 | 990 | 13.72 |
| Inferior Opercular Frontal | R |  |  | 920 |  |
| Insula | R |  |  | 865 |  |
| Inferior Triangular Frontal | R |  |  | 408 |  |
| Putamen | R |  |  | 393 |  |
| Caudate | R |  |  | 327 |  |
| Inferior Tri Frontal  *Cluster Sub Regions* | L | 9 | 50, 6, 30 | 1214 | 9.70 |
| Inferior Opercular Frontal | L |  |  | 803 |  |
| Precentral Gyrus | L |  |  | 790 |  |
| Middle Frontal | L |  |  | 500 |  |
| Insula | L |  |  | 474 |  |
| Middle Occipital  *Cluster Sub Regions* | R | 40 | 42,-56,-18 | 842 | 8.48 |
| Fusiform | R |  |  | 672 |  |
| Inferior Parietal | R |  |  | 604 |  |
| Cerebelum | R |  |  | 478 |  |
| Inferior Temporal | R |  |  | 339 |  |
| SupraMarginal | R |  |  | 269 |  |
| SMA  *Cluster Sub Regions* | L | 6 | -8,11,68 | 840 | 6.59 |
| SMA | R |  |  | 594 |  |
| Middle Cingulum | R |  |  | 361 |  |
| Superior Frontal | R,L |  |  | 335, 249 |  |
| Inferior Parietal  *Cluster Sub Regions* | L | 40 | -30,-56,40 | 1314 | 6.49 |
| Superior Parietal | L |  |  | 430 |  |
| Middle Temporal  *Cluster Sub Regions* | R | 22 | 65,-45,26 | 686 | 6.28 |
| Superior Temporal | R |  |  | 358 |  |
| **Week 12 Deactivation** |  |  |  |  |  |
| Precuneus  *Cluster Sub Regions* | L | 31 | 39,-21,27 | 576 | 9.08 |
| Precuneus | R |  |  | 438 |  |
| Posterior Cingulum | L |  |  | 414 |  |
| Middle Cingulum | L,R |  |  | 358, 234 |  |
| Medial Superior Frontal  *Cluster Sub Regions* | L | 10 | -5,48,-11 | 835 | 8.11 |
| Anterior Cingulum | L,R |  |  | 646, 236 |  |
| Medial OFC | L,R |  |  | 604, 422 |  |
| Superior Frontal | L |  |  | 447 |  |
| Medial Superior Frontal  *Cluster Sub Regions* | R |  |  | 429 |  |
| Middle Frontal | L |  |  | 319 |  |

**Table S2.** Areas psycho-physiologically correlated with the dACC during Stroop task.

| Region | Hem | BA | x,y,z | Cluster size | peak *t* Value |
| --- | --- | --- | --- | --- | --- |
| **Baseline Correlation** | - | - | - | - | - |
| **Baseline Anti Correlation** |  |  |  |  |  |
| Precuneus  *Cluster Sub Regions* | L |  | 9,-24,54 | 662 | 8.86 |
| Precuneus | R |  |  | 560 |  |
| Middle Cingulum | L |  |  | 547 |  |
| Calcarine | L,R |  |  | 375, 209 |  |
| Lingual | R |  |  | 266 |  |
| Posterior Cingulate | L |  |  | 232 |  |
| Middle Cingulum | R |  |  | 209 |  |
| Superior Temporal gyrus | R |  | 50,-33,27 | 455 | 6.81 |
| Superior Medial Frontal  *Cluster Sub Regions* | R |  | 21,56,-14 | 436 | 6.07 |
| Superior Medial Frontal | L |  |  | 337 |  |
| Anterior Cingulum | L,R |  |  | 307, 224 |  |
| Superior OFC | R |  |  | 152 |  |
| Postcentral gyrus  *Cluster Sub Regions* | R |  | 36,-38,57 | 509 | 4.73 |
| Primary Motor | R |  |  | 437 |  |
| **Week 12 Correlation** | - | - | - | - | - |
| **Week 12 Anti Correlation** |  |  |  |  |  |
| Inferior Parietal  *Cluster Sub Regions* | L |  | 29,-74,35 | 1214 | 10.60 |
| Postcentral gyrus | R,L |  |  | 1210, 717 |  |
| Precuneus | R,L |  |  | 1103, 966 |  |
| Inferior parietal lobule | R |  |  | 1097 |  |
| Primary motor | R |  |  | 1077 |  |
| Lingual gyrus | R,L |  |  | 1010, 897 |  |
| Cuneus | R |  |  | 961 |  |
| Calcarine | L,R |  |  | 893, 835 |  |
| Superior Parietal | L,R |  |  | 890, 831 |  |
| SMA | R,L |  |  | 806, 806 |  |
| Middle Frontal | R |  |  | 791 |  |
| Superior Occipital | R |  |  | 753 |  |
| Superior Frontal | R |  |  | 712 |  |
| Middle Occiptal | R,L |  |  | 614, 583 |  |
| Cerebelum 4, 5 | L |  |  | 529 |  |
| SupraMarginal Gyrus | R |  |  | 494 |  |
| Cuneus | L |  |  | 483 |  |
| Fusiform | R,L |  |  | 472, 302 |  |
| Angular Gyrus | R |  |  | 419 |  |
| Precentral | L |  |  | 353 |  |
| Cerebelum 6 | L |  |  | 352 |  |
| Superior Occipital | L |  |  | 331 |  |
| Middle Temporal | R,L |  |  | 246, 238 |  |
| Middle Cingulum | R,L |  |  | 240, 187 |  |
| Superior Frontal | L |  |  | 225 |  |
| Inferior Tri frontal | R |  |  | 224 |  |
| Inferior Temporal | R |  |  | 195 |  |

BA, Brodmann’s Area; Hem, hemisphere; L,R indicates bilateral activation with corresponding cluster sizes separated by a comma; x,y,z refer to Montreal Neurological Institute coordinates. OFC, Orbitofrontal Cortex; SMA, Supplementary Motor Area All regions are significant at a cluster level threshold of p<.05 FWE cluster level corrected.

**Table S3.** BOLD Contrast Results of Study Completers compared to Study Non-Completers.

| Region | Hem | x,y,z | Cluster size | peak *t* Value |
| --- | --- | --- | --- | --- |
| **Non-completers>Completers*** |  |  |  |  |
| Putamen | R | 15,6,5 | 655 | 5.40 |
| *Cluster Sub Regions* |  |  |  |  |
| Thalamus | R |  | 180 |  |
| Globus Pallidus | R |  | 85 |  |
| Caudate nucleus | R |  | 85 |  |
| Insula | R |  | 49 |  |
| Insula | L | -38,5,-14 | 550 | 4.64 |
| *Cluster Sub Regions* |  |  |  |  |
| Middle Frontal Gyrus | L |  | 434 |  |
| Inferior Frontal Gyrus | L |  | 380 |  |
| Putamen | L |  | 301 |  |
| Precentral Gyrus | L |  | 222 |  |
| Superior Temporal Gyrus | L |  | 204 |  |
| Uncus | L |  | 83 |  |
| Middle Occipital | L | -30,-65,45 | 459 | 4.32 |
| *Cluster Sub Regions* |  |  |  |  |
| Inferior Parietal | L |  | 152 |  |
| Superior Occipital | L |  | 116 |  |
| Superior Parietal | L |  | 108 |  |
| Angular Gyrus | L |  | 89 |  |
| **Completers> Non-completers**** | - | - | - | - |

* Non-Completers>Completers results p<.05 FWE cluster level corrected.

** Nothing in Completers> Study Non-Completers survived cluster level correction

**Table S4 Glutamate Measurement in Dorsal Anterior Cingulate Cortex**

| Metabolite | Baseline Non Completers | Baseline Completers | *t* Value | *p* Value |
| --- | --- | --- | --- | --- |
| Glx/Cr | .694 ± .115 | .705 ± .089 | .243 | .811 |
| CRLB±SD  (CRLB Range) | 7.3±1.8%  (5.5–12.1%) | 7.1±1.7%  (5.8–11.6%) |  |  |

*Note.* Cr, creatine, CRLB, Cramer-Rao Lower Bounds; Glx, glutamate + glutamine;

There were no significant differences in the raw amplitude of Cr between groups t(19)=.517, p=.611. There were no significant differences between non-completers and completers in metabolite peak line width, CRLBs, gray matter voxel content, or white matter voxel content.

Independent samples t-test conducted on baseline Glx/Cr for study non-completers (N=10) compared to baseline study completers (N=11).


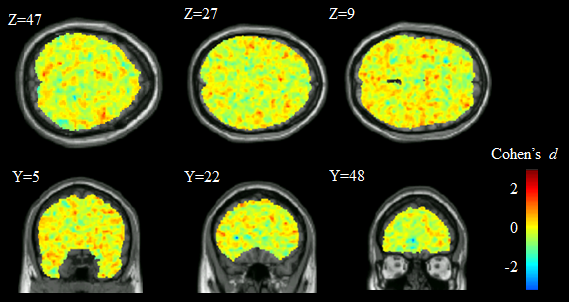


**Figure S1.** Whole brain effect size (Cohen’s *d*) of Post>Pre-drug BOLD contrast. Although only eleven subjects completed the study, many parts of the contrast have a large effect size (i.e. greater than 0.8) indicated by orange and teal values for both sides of the t-test respectively.
